# Gut Microbiota of *Drosophila subobscura* Contributes to Its Heat Tolerance and Is Sensitive to Transient Thermal Stress

**DOI:** 10.3389/fmicb.2021.654108

**Published:** 2021-05-06

**Authors:** Angélica Jaramillo, Luis E. Castañeda

**Affiliations:** Programa de Genética Humana, Instituto de Ciencias Biomédicas, Facultad de Medicina, Universidad de Chile, Santiago, Chile

**Keywords:** bacterial microbiota, climate change, fruit fly, heat stress, stress resistance

## Abstract

The gut microbiota can contribute to host physiology leading to an increase of resistance to abiotic stress conditions. For instance, temperature has profound effects on ectotherms, and the role of the gut microbiota on the thermal tolerance of ectotherms is a matter of recent research. However, most of these studies have been focused on single static temperatures instead of evaluating thermal tolerance in a wide range of stressful temperatures. Additionally, there is evidence supporting that the gut microbiota is sensitive to environmental temperature, which induces changes in its composition and diversity. These studies have evaluated the effects of thermal acclimation (>2 weeks) on the gut microbiota, but we know little about the impact of transient thermal stress on the composition and diversity of the gut microbiota. Thus, we investigated the role of the gut microbiota on the heat tolerance of *Drosophila subobscura* by measuring the heat tolerance of conventional and axenic flies exposed to different heat stressful temperatures (35, 36, 37, and 38°C) and estimating the heat tolerance landscape for both microbiota treatments. Conventional flies exposed to mild heat conditions exhibited higher thermal tolerance than axenic flies, whereas at higher stressful temperatures there were no differences between axenic and conventional flies. We also assessed the impact of transient heat stress on the taxonomical abundance, diversity, and community structure of the gut microbiota, comparing non-stressed flies (exposed to 21°C) and heat-stressed flies (exposed to 34°C) from both sexes. Bacterial diversity indices, bacterial abundances, and community structure changed between non-stressed and heat-stressed flies, and this response was sex-dependent. In general, our findings provide evidence that the gut microbiota influences heat tolerance and that heat stress modifies the gut microbiota at the taxonomical and structural levels. These results demonstrate that the gut microbiota contributes to heat tolerance and is also highly sensitive to transient heat stress, which could have important consequences on host fitness, population risk extinction, and the vulnerability of ectotherms to current and future climatic conditions.

## Introduction

The gut microbiota influences multiple features of the host’s biology, including nutrient acquisition, immune response, metabolism, behavior, and life history traits (Broderick and Lemaitre, 2012; Douglas, 2018a; Hoye and Fenton, 2018). In general, the gut microbiota influences the phenotypic variations exhibited by host organisms, which can contribute to speeding up their adaptive responses under changing and fluctuating environments (Alberdi et al., 2016; Macke et al., 2017; Romano, 2017). However, environmental variations, ranging from benign to stressful conditions, also impact the composition and diversity of the gut microbiota, altering its contribution to host phenotypic variability and modifying the functional relationship between hosts and the gut microbiota (Sepulveda and Moeller, 2020).

Among multiple environmental factors, temperature has profound effects on the physiology, behavior, and performance of ectotherms because the body temperature of ectotherms is influenced by the environmental temperature (Angilletta, 2009). The ongoing climate change is expected to impose strong selection pressures on the heat tolerance of ectotherms (Hoffmann and Sgrò, 2011), and the gut microbiota can contribute to host thermal tolerance (Kokou et al., 2018). Indeed, recent evidence demonstrates that the microbiota impacts on the thermal performance of ectotherm species (Renoz et al., 2019; Sepulveda and Moeller, 2020). For instance, obligatory endosymbionts contribute to aphid performance at high temperatures (Dunbar et al., 2007; Zhang et al., 2019), whereas facultative endosymbionts also confer tolerance to high temperature in aphids (Montllor et al., 2002; Russell and Moran, 2006) and *Drosophila* (Gruntenko et al., 2017). Additionally, it has been demonstrated that the gut microbiota also influences the cold and heat tolerance of ectotherm species (Ziegler et al., 2017; Henry and Colinet, 2018; Kokou et al., 2018; Moghadam et al., 2018; Raza et al., 2020). However, most of these studies have been focused on single static temperatures instead of evaluating thermal tolerance in a wide range of stressful temperatures. This aspect is very important because there is evidence supporting that heat tolerance depends on the intensity and duration of the thermal challenge, indicating that heat tolerance is strongly influenced by the methodology employed (Rezende et al., 2011; Castañeda et al., 2019; Semsar-Kazerouni et al., 2020). Therefore, the description of the heat tolerance landscape provides a better description of the thermal tolerance of ectotherms exposed to high, stressful temperatures (Rezende et al., 2014).

On the other hand, several studies have also explored the impact of temperature on the host microbiota, indicating that the gut microbiota is sensitive to environmental temperature (Wernegreen, 2012; Sepulveda and Moeller, 2020). Temperature induces changes in the composition and diversity of the gut microbiota, which could have important consequences on host phenotype and fitness (Wernegreen, 2012; Alberdi et al., 2016). For example, small ectotherms reared at high temperatures show an increase in the abundance of bacteria belonging to the phylum Proteobacteria (Li et al., 2018; Moghadam et al., 2018; Horváthová et al., 2019). Indeed, *Drosophila melanogaster* flies acclimated in warm conditions showed a higher abundance of *Acetobacter* bacteria (Proteobacteria) and a lower abundance of *Leuconostoc* bacteria (Firmicutes) in comparison to cold-acclimated flies (Moghadam et al., 2018). On the other hand, several studies have demonstrated that bacterial diversity and richness decrease when hosts are exposed to warm conditions (Kokou et al., 2018; Moghadam et al., 2018). However, most of these studies have used thermal acclimation (i.e., >2 weeks) to evaluate changes in the gut microbiota composition, but we know little about the impact of transient thermal stress on the composition and diversity of the gut microbiota.

Therefore, to evaluate the role of the gut microbiota on heat tolerance, we compared the heat tolerance landscape between conventional (non-manipulated) and axenic (germ-free) flies exposed to different heat stressful temperatures (35, 36, 37, and 38°C). We also assessed the impact of transient heat stress on the taxonomical abundance, diversity, and community structure of the gut microbiota, comparing non-stressed and heat-stressed flies (exposed to 21 and 34°C, respectively). This experiment should provide new findings in order to understand the thermal sensitivity of the gut microbiota to sudden changes of temperature (e.g., heatwaves). We used *Drosophila subobscura* as the study model because, since its introduction in Chile at the end of the 1970s (Brncic et al., 1981), this species has shown a rapid expansion of its distribution range and shows evidence of thermal adaptation in several phenotypic traits (Huey et al., 2000; Gilchrist et al., 2008; Castañeda et al., 2013, 2015). Therefore, it is interesting to explore the relationship between temperature and the gut microbiota in *D. subobscura* in order to have a better understanding of how ectotherm species respond to thermal challenges and adapt to new environments.

## Materials and Methods

### *Drosophila* Sampling and Maintenance

Adult *D. subobscura* flies were collected at the locality of Valdivia (southern Chile: 39°48′ S, 73°14′ W) and separated by sex. Females were individually placed in plastic vials with David’s killed-yeast *Drosophila* medium (David, 1962) to establish isofemale lines. At the next generation, 100 isofemale lines were randomly selected, and adult flies were dumped into an acrylic cage to set up one large outbred population, which was maintained in a climatic chamber (Bioref, Pitec, Chile) at 21 ± 1°C and a 12L/12D photoperiod. The maintenance conditions were similar in all experiments, and the population cage was maintained on a discrete generation, controlled larval density regime (Castañeda et al., 2015).

### Preparation of Axenic and Conventional Flies

Axenic (germ-free) flies were obtained by using dechorionated eggs (Koyle et al., 2016). Eggs (≤18 h old) were collected from Petri dishes containing fly media placed within the population cage and dechorionated as follow: three washes with 0.5% hypochlorite sodium solution per 2 min wash, three washes with 70% ethanol solution per 2 min wash, and three washes with autoclaved water per 2 min wash. Dechorionated eggs were transferred to 50-ml Falcon tubes containing autoclaved *Drosophila* media at a density of 50 eggs/tube. The procedure to obtain axenic flies was performed under sterile conditions in a flow laminar chamber. For conventional (non-manipulated microbiota) flies, the eggs were collected from the same Petri dishes used previously, washed four times with autoclaved water, and transferred to 50-ml Falcon tubes containing autoclaved *Drosophila* media at a density of 50 eggs/tube.

Elimination of bacteria in axenic flies was corroborated by testing the amplification of bacterial DNA. Medium samples and 10 flies were randomly collected from tubes containing axenic flies. From both types of samples, DNA was extracted using the GeneJet kit (Thermo Fisher) following the protocol of extracting DNA from Gram-negative and Gram-positive bacteria. Then, a PCR was performed to amplify the bacterial DNA using specific primers for the 16S ribosomal RNA (rRNA) gene: 341F (5′-CCT ACG GGN GGC WGC AG-3′) and 805R (5′-GGA CTA CHV GGG TWT CTA AR-3′) (Fadrosh et al., 2014). The PCR mix contained 0.02 U DNA polymerase (Invitrogen), 1 × PCR buffer, 0.2 mM deoxynucleotide triphosphate (dNTP), 1 μM of each primer, 0.5 μM MgCl_2_, and 0.5 μl template DNA. The PCR cycle conditions were set up following the recommendations of Caporaso et al. (2010b): denaturation at 94°C for 3 min; 35 amplification cycles at 94°C for 45 s, 52°C for 1 min, and 72°C for 70 s; and a final extension at 72°C for 10 min. PCR products were loaded on a 2% agarose gel stained with Sybr Safe (Invitrogen). DNA extractions from conventional flies were used as bacteria-positive controls. Thus, effective bacterial elimination was considered effective when no amplification band was visualized in the agarose gel. If flies considered as axenic resulted positive for bacterial amplification, they were discarded.

### Heat Tolerance of Axenic and Conventional Flies

Axenic and conventional virgin flies of both sexes at the age of 4 days were individually placed in capped 5-ml glass vials, which were attached to a rack with capacity to contain 60 capped vials. In each rack, we placed 15 axenic females, 15 axenic males, 15 conventional females, and 15 conventional males. Each rack was immersed in a water tank at a specific static temperature: 35, 36, 37, and 38°C. The temperature (±0.1°C) was controlled by a heating unit (model ED, Julabo Labortechnik, Seelbach, Germany). Each static assay was photographed using a high-resolution camera (D5100, Nikon, Tokyo, Japan) and photos were taken every 3 s. The photos for each assay were collated in a video file, which was visualized to score the knockdown time measured as the time at which each fly ceased to move (Castañeda et al., 2019).

### Heat Stress Exposure

Petri dishes with fly medium were placed within the population cage for collecting eggs. Eggs (≤18 h old) were transferred into vials at a density of 40 eggs/vial. After eclosion, virgin flies were separated by sex and transferred to new vials. At the age of 4 days, 100 females and 100 males were transferred to empty vials at a density of 25 flies/vial and the vials closed with moistened stoppers to avoid fly desiccation. The vials were split into two groups: non-stressed and heat-stressed flies. Non-stressed flies were transferred into a climatic chamber (Bioref, Pitec, Chile) at 21 ± 1°C for 3 h, whereas heat-stressed flies were placed in a water bath at 34°C for 1 h; temperature (±0.1°C) was controlled by a heating unit (model ED, Julabo Labortechnik, Seelbach, Germany). This temperature was chosen because it has been previously used to induce thermal stress in *D. melanogaster* (Hoffmann et al., 2003) and *D. subobscura* (Calabria et al., 2012). Then, heat-stressed flies were transferred into a climatic chamber (Bioref, Pitec, Chile) at 21 ± °1°C for 2 h for recovery from heat stress (no fly died after stress).

### DNA Extraction and Amplicon Sequencing

Flies of each thermal stress treatment (non-stressed and heat-stressed flies) and sex were pooled into groups of five flies each: 10 pools of non-stressed females, 10 pools of non-stressed males, 10 pools of heat-stressed females, and 10 pools of heat-stressed males. To eliminate superficial bacteria, each pool was given three washes with 0.5% hypochlorite sodium solution for 2 min each wash, three washes with 70% ethanol solution for 2 min each wash, and three washes with autoclaved water for 2 min each wash. Then, each pool was transferred into a Petri dish with sterile 1 × PBS solution, where the intestines of flies were removed and transferred into Eppendorf tubes with ice-cold sterile 1 × PBS solution.

Genomic DNA was extracted from pooled guts using the GeneJet kit (Thermo Fisher) following the protocol for Gram-negative and Gram-positive bacteria. Then, the V3–V4 hypervariable region of the 16S rRNA gene was amplified using a dual-indexing approach according to Fadrosh et al. (2014). Amplicon PCR was performed using modified 341F and 805F primers, which contained: (1) a linker sequence to bind amplicons to the Nextera XT DNA indices; (2) a 12-bp barcode sequence to multiplex samples; (3) a 0–5 bp “heterogeneity spacer” to increase the heterogeneity of amplicon sequences; and (4) 16S rRNA gene universal primers (Supplementary Table 1). The amplicon PCR mix had a final volume of 12.5 μl: 6.5 μl ultrapure water, 5 μl 2 × Hot Start PCR Master Mix (Invitrogen), 0.25 μl 1 μM forward primer, 0.25 μl 1 μM reverse primer, and 0.5 μl template genomic DNA. The amplicon PCR cycle conditions were set up as follows: denaturation at 94°C for 3 min, 35 amplification cycles at 94°C for 45 s, 52°C for 1 min, 72°C for 70 s, and a final extension at 72°C for 10 min. Amplified reactions were purified using an enzyme mix (exonuclease I and Fast AP, Invitrogen) to eliminate free primers and dNTPs and then loaded on a 2% agarose gel stained with Sybr Safe (Invitrogen) to visualize the PCR products.

The PCR products were quantified by fluorescence using the Quan-iT PicoGreen dsDNA kit (Invitrogen), and then all samples were standardized at the lowest DNA concentration samples (7.78 ng/μl). The primer design allowed multiplexing 23 samples into two different sets of Nextera XT DNA indices (Illumina Corporation, San Diego, CA, United States). The index PCR had a final volume of 50 μl: 5 μl amplicon PCR, 5 μl indices (N701 and S502 for library 1; N707 and S506 for library 2), 25 μl 2 × KAPA Taq HotStart DNA Polymerase (Invitrogen), and 10 μl ultrapure water. The index PCR cycle conditions were set up as follows: denaturation at 94°C for 3 min, eight amplification cycles at 95°C for 30 s, 55°C for 30 s, 72°C for 30 s, and a final extension at 72°C for 5 min. The PCR products were cleaned using the AMPure XT Bead kit (Beckman Coulter, Brea, CA, United States) and quantified using the Qubit Fluorometer and Qubit dsDNA HS assay kit. Library 1 had a concentration of 37.2 ng/μl and library 2 a concentration of 46.2 ng/μl; both libraries were diluted at a concentration of 4 nM. Libraries were sequenced using an Illumina MiSeq sequencer (Illumina, San Diego, CA, United States) and the MiSeq Reagent v3 (600 cycles). Sequencing was performed at the AUSTRAL-omics Sequencing Core Facility at Universidad Austral de Chile.

### Metabarcoding Analysis

After sequencing, 3,061,220 sequences were obtained. Raw sequence quality was inspected using FastQC (Andrews, 2010) and then filtered for a *Q* value higher than 28 and sequences longer than 150 bp using the script Reads_Quality_Length_distribution.pl (Bálint et al., 2014). Forward and reverse filtered sequences were paired using PANDASeq with a minimum overlap of 5 bp (Masella et al., 2012). Paired-end sequences were trimmed to remove forward/reverse barcodes, heterogeneity spacers, and 16S rRNA gene primers. Quality-filtered and trimmed sequences were analyzed using QIIME v1.9.1 (Caporaso et al., 2010a). An open-reference OTU-picking strategy was used to generate operational taxonomic units (OTUs) using the *usearch* v6.1 algorithm to cluster OTUs at 97% of nucleotide identity. Taxonomy assignment was performed using the *uclust* method (Edgar, 2010) against the Greengenes 16S rRNA gene database at 97% pairwise identity (version 13.8; Mcdonald et al., 2012) as database reference. Finally, representative OTU sequences were aligned using PyNast and used to build a phylogenetic tree using FastTree. After this procedure, we retained 1,559,937 sequences assigned to 1,263 OTUs. After this, we performed two filtering steps: (1) remove mitochondrial-, chloroplast-, *Spiroplasma*-, and *Wolbachia*-related sequences and (2) remove OTUs comprising less than 100 sequences. The retained sequences (total = 1,538,400, range = 746–59,210) and the OTU number (total = 135) by sample are reported in Supplementary Table 2. For diversity analyses, the samples were rarified at 12,000 sequences according to the rarefaction curve (Supplementary Figure 1), which resulted in the removal of four samples (1FDRD, 2FDRD, 1FBRB, and 2FBRB; see Supplementary Table 2).

### Statistical Analyses

#### Gut Microbiota and Heat Tolerance

Knockdown time was transformed to log_10_ and analyzed with a linear model, which included sex, microbiota treatment, and assayed temperatures as the explanatory variables. We also tested the differences between the survival curves of axenic and conventional flies at each static assay with the G-rho family test (log-rank test) using the *survival* R package (Therneau, 2020); the survival curves were plotted using the *survminer* R package (Kassambara et al., 2020).

#### Gut Microbiota Composition and Diversity

We analyzed the effects of heat stress, sex, and its interaction on the bacterial abundance, diversity indices, and community structure of the gut microbiota. Firstly, relative abundance at the phylum and family taxonomical levels were obtained using the *phyloseq* (Mcmurdie and Holmes, 2013) and *microbiome* (Lahti et al., 2017) packages for R, and then relative abundances were compared using a generalized linear model (GLM) assuming a quasi-binomial distribution. Secondly, we analyzed the OTU relative abundances between the non-stressed and heat-stressed flies for each sex using the package *DESeq2* for R (Love et al., 2014). DESeq2 uses a negative binomial model for count data, taking into account the zero-skewed distribution of the microbiome dataset. Significant differences between groups in OTU relative abundance were considered when the adjusted *P* value [false discovery rate (FDR) correction] was lower than 0.05. Thirdly, OTU richness and Shannon diversity were estimated using the *microbiome* package for R (Lahti et al., 2017), whereas the phylogenetic diversity was estimated using QIIME. Diversity indices were analyzed using a two-way ANOVA, and *a posteriori* comparisons were performed using a Bonferroni *t* test. Finally, we estimated the weighted UniFrac distances among samples using QIIME, which was used as input to compare the bacterial community structure between thermal stress treatment (non-stressed and heat-stressed flies) and sexes (females and males). Bacterial community analysis was performed through a permutational multivariate analysis of variance (PERMANOVA) using the *vegan* package for R (Oksanen et al., 2020).

All statistical analyses were performed using R version 4.0.3 (R Core Team, 2020) and RStudio version 1.3.959 (RStudio Team, 2020), and plots were made using the *ggpubr* (Kassambara, 2020a) and *rstatix* (Kassambara, 2020b) packages for R.

## Results

### Effect of the Gut Microbiota on Heat Tolerance

**FIGURE 1:**
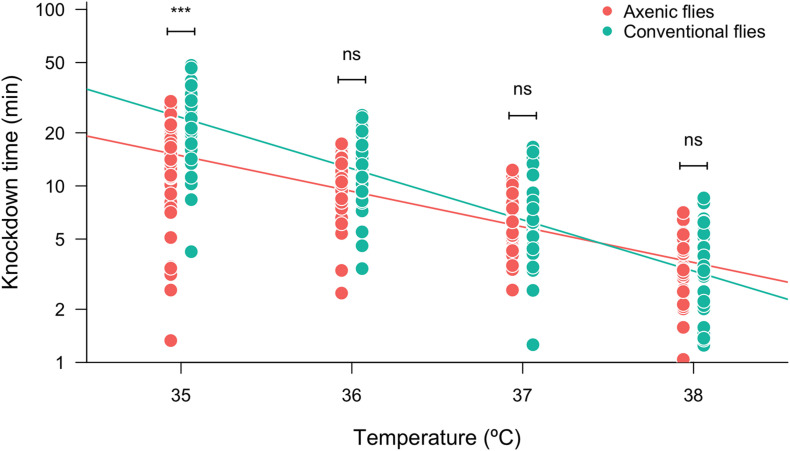
Heat tolerance (measured as log_10_-transformed knockdown time) of axenic and conventional flies estimated at different assayed temperatures. Symbols above box plots denote non-significant (ns) or significant differences between the axenic and conventional flies obtained from a linear model (***P < 0.001).

To evaluate the role of the gut microbiota on the heat tolerance landscape, we compared the heat knockdown time between the conventional (non-manipulated) and axenic (germ-free) flies exposed to different heat stressful temperatures. We found that knockdown time was affected by the assayed temperature: the higher the assayed temperature, the shorter the knockdown time (Figure 1 and Table 1). The heat tolerance landscape was different between the axenic and conventional flies because we found significant differences in the intercept (*P* = 0.0158) and a significant interaction between the assayed temperatures and microbiota treatments (slope: *P* = 0.0166) (Figure 1 and Table 1). Specifically, we found that the knockdown time between the axenic and conventional flies was significantly different at 35°C (*F*_1,472_ = 53.81, *P* < 0.0001), where conventional flies showed longer knockdown times than the axenic flies. Conversely, heat tolerance did not differ between the microbiota treatments at temperatures higher than 35°C. On the other hand, the knockdown temperature was not affected by sex or by the interactions between sex and the other factors (Table 1). Additionally, we evaluated the effect of the gut microbiota on the knockdown (survival) curves (Supplementary Figure 2), and we found that the axenic and conventional flies showed different shape curves at 35°C (*P* < 0.0001; Supplementary Figure 2A) and at 36°C (*P* = 0.033; Supplementary Figure 2B), but not at 37°C (*P* = 0.62; Supplementary Figure 2C) and 38°C (*P* = 0.56; Supplementary Figure 2D).

**TABLE 1:** Results of linear model testing effects of the assayed temperature, microbiota treatment, and sex on the heat tolerance (measured as the knockdown time in static assays) of *Drosophila subobscura*.

Effect	Estimate	DF (num, den)	*t* value	*P* value
Intercept	11.9320	1,472	17.673	<0.0001
Temperature	−0.3015	1,472	−16.306	<0.0001
Microbiota treatment	−2.3124	1,472	−2.422	0.0158
Sex	−0.7708	1,472	−0.807	0.4199
Temperature/microbiota	0.0628	1,472	2.403	0.0166
Temperature/sex	0.0225	1,472	0.860	0.3904
Microbiota/sex	−2.0009	1,472	−1.482	0.1391
Temperature/microbiota/sex	0.0513	1,472	1.386	0.1664

### Effect of Transient Heat Stress on the Gut Microbiota Composition

**FIGURE 2:**
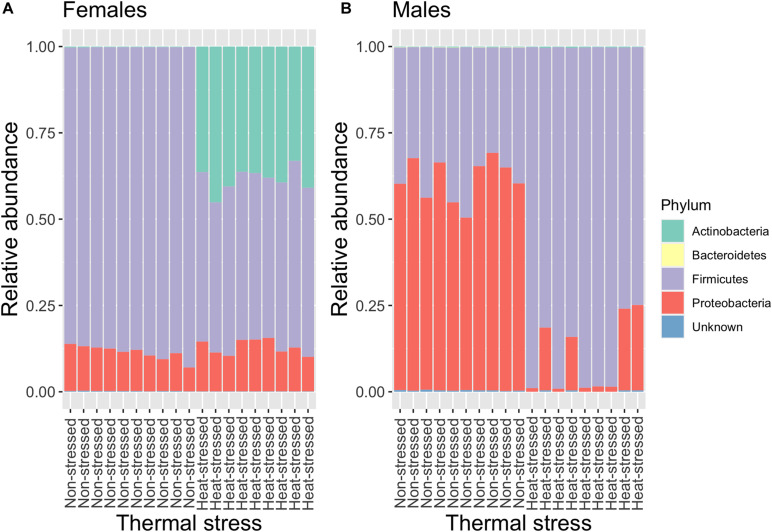
Relative abundance of the bacterial composition (phylum) of the gut microbiota of non-stressed (flies exposed to 21°C) and heat-stressed (flies exposed to 34°C for 1 h) females **(A)** and males **(B)** of *Drosophila subobscura*. Each *color* represents a bacterial phylum.

We analyzed the gut microbiota composition of the flies exposed to a non-stressful temperature (21°C, non-stressed flies) and the flies exposed to a stressful thermal condition (34°C, heat-stressed flies). As results from this experiment, we found that the gut microbiota of *D. subobscura* was dominated by bacteria belonging Actinobacteria (mean relative frequency ± SE = 0.09 ± 0.03), Bacteroidetes (mean relative frequency ± SE = 0.0002 ± 0.00004), Firmicutes (mean relative frequency ± SE = 0.66 ± 0.04), and Proteobacteria (mean relative frequency ± SE = 0.24 ± 0.04). In general, we found that the relative abundance of these phyla depended on heat stress and the flies’ sex (Figure 2). Actinobacteria increased their abundances from 0.2% in non-stressed females to 38.5% in heat-stressed females (GLM: *t* = −16.14, *P* = 9.6 × 10^–12^) (Supplementary Figure 2A), whereas the increase was more moderated in male flies (GLM: *t* = −2.49, *P* = 0.02) (Supplementary Figure 2A). The relative abundance of Bacteroidetes showed a significant interaction between heat stress and sex (GLM: *t* = 3.23, *P* = 0.003), with males showing an important reduction of Bacteroidetes abundance between non-stressed and heat-stressed flies (GLM: *t* = 5.58, *P* = 3.3 × 10^–5^) (Supplementary Figure 2B) in comparison to female flies (GLM: *t* = 2.26, *P* = 0.04) (Supplementary Figure 2B). Similarly, Proteobacteria abundance analysis showed a significant interaction between heat stress and sex (GLM: *t* = 7.77, *P* = 4.8 × 10^–9^) (Supplementary Figure 2C): females showed similar abundance between non-stressed and heat-stressed flies (GLM: *t* = −1.69, *P* = 0.11), whereas heat stress induced an important reduction of Proteobacteria abundance in males (GLM: *t* = 7.92, *P* = 4.2 × 10^–5^). On the other hand, Firmicutes abundance showed an interaction response between heat stress and sex (GLM: *t* = −14.38, *P* = 5.2 × 10^–16^) (Supplementary Figure 2D): females exposed to heat stress displayed a decrease in Firmicutes abundance from 88.4 to 48.6% (GLM: *t* = 29.18, *P* = 5.8 × 10^–16^), whereas heat stress induced an increase of Firmicutes abundance from 38.2% in non-stressed males to 89.4% in heat-stressed males (GLM: *t* = −7.99, *P* = 3.7 × 10^–7^).

Among the most abundant bacterial families (abundance higher than 1%) associated with the gut of *D. subobscura*, we found Acetobacteraceae (mean relative frequency ± SE = 0.19 ± 0.03), Dermabacteraceae (mean relative frequency ± SE = 0.09 ± 0.03), Halomonadaceae (mean relative frequency ± SE = 0.02 ± 0.003), Lactobacillaceae (mean relative frequency ± SE = 0.45 ± 0.04), and Leuconostocaceae (mean relative frequency ± SE = 0.21 ± 0.05). Particularly, we focused on the relative abundances of acetic acid and lactic acid bacteria (AAB and LAB, respectively). We found a significant interaction between heat stress and sex for AAB (Acetobacteraceae) abundance (GLM: *t* = 6.97, *P* = 4.8 × 10^–8^) (Supplementary Figure 3A), which is explained because, whereas AAB abundance increased in heat-stressed females compared to non-stressed females (GLM: *t* = −7.20, *P* = 1.5 × 10^–6^), non-stressed males showed a higher abundance than the heat-stressed males (GLM: *t* = 6.05, *P* = 1.3 × 10^–5^). On the other hand, LAB families exhibited contrasting responses to heat stress. Lactobacillaceae abundance showed a significant interaction between heat stress and sex (GLM: *t* = −2.80, *P* = 0.008) (Supplementary Figure 3B): heat-stressed females showed a lower abundance of Lactobacillaceae than the non-stressed females (GLM: *t* = 17.93, *P* = 1.8 × 10^–12^), whereas non-stressed and heat-stressed males showed similar Lactobacillaceae abundances (GLM: *t* = 1.67, *P* = 0.11). On the other hand, Leuconostocaceae abundance also showed a significant interaction between heat stress and sex (GLM: *t* = −5.45, *P* = 4.4 × 10^–6^) (Supplementary Figure 3C): non-stressed females showed a higher Leuconostocaceae abundance than the heat-stressed females (GLM: *t* = 2.37, *P* = 0.03), whereas heat-stressed males harbored a higher Leuconostocaceae abundance than the non-stressed males (GLM: *t* = −4.85, *P* = 0.0001).

**FIGURE 3:**
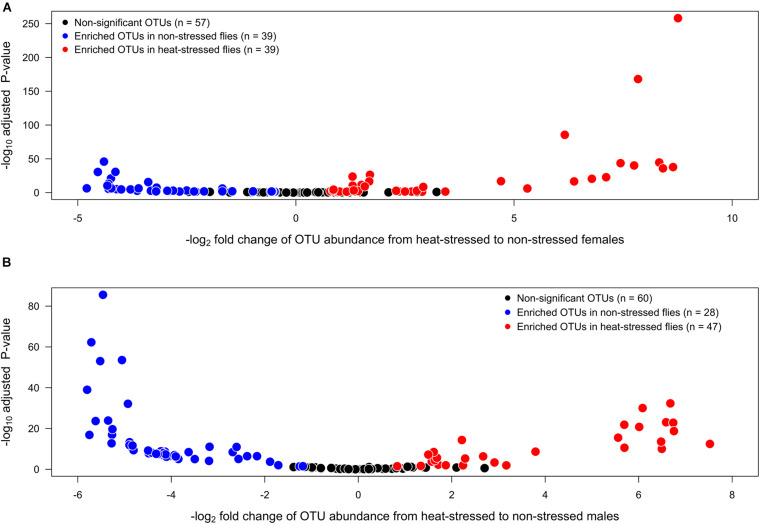
Volcano plots illustrating the bacterial operational taxonomic units (OTUs) that show significantly higher abundances in non-stressed (exposed to 21°C, blue circles) or heat-stressed (exposed to 34°C, red circles) flies for each sex: **(A)** females and **(B)** males of *Drosophila subobscura*. OTUs that exhibit similar abundances in non-stressed and heat-stressed flies are represented by black circles.

We also found that heat stress induced changes in the abundance of individual OTUs. We found a total of 135 OTUs in the gut of *D. subobscura* whose identity did not differ between females and males. However, when we compared the OTU abundances between the non-stressed and heat-stressed flies, we found that each sex showed specific responses. The analysis for female flies (Figure 3A) showed that, after the heat stress, 39 OTUs significantly decreased their abundances (blue circles) and 39 OTUs significantly increased their abundances (red circles), whereas 57 OTUs did not change their abundances between the non-stressed and heat-stressed females (black circles). On the other hand, the analysis for males (Figure 3B) showed that 28 OTUs significantly decreased their abundances in the non-stressed females (blue circles), whereas 47 OTUs significantly increased their abundances in the heat-stressed females (red circles); 60 OTUs did not change their abundances between the non-stressed and heat-stressed females (black circles). We also analyzed the co-occurrence of OTUs across sexes: only five OTUs showed increased abundances in the non-stressed females and males (33 OTUs increased exclusively in non-stressed females and 19 OTUs increased exclusively in non-stressed males). On the other hand, we found that 31 OTUs increased their abundances in the heat-stressed females and males (four OTUs increased exclusively in heat-stressed females and 15 OTUs increased exclusively in heat-stressed males).

### Effect of Transient Heat Stress on the Gut Microbiota Diversity

**FIGURE 4:**
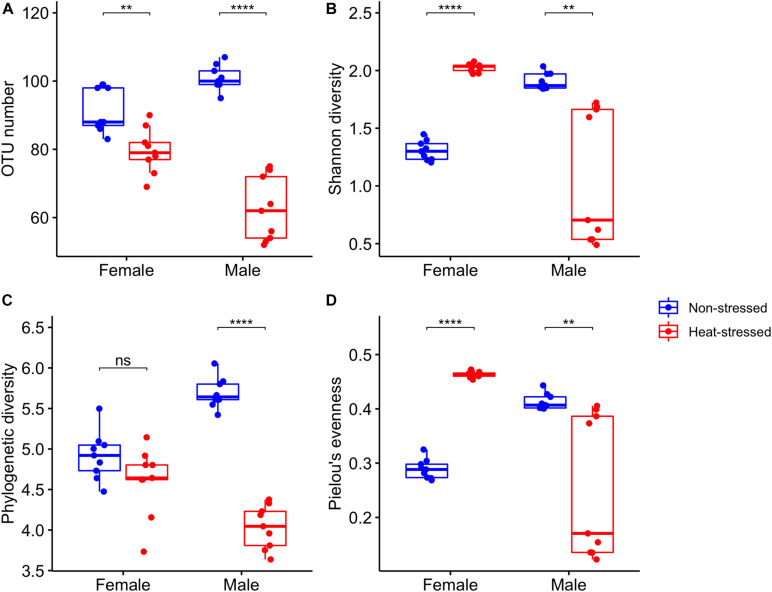
Bacterial diversity indices estimated for the gut microbiota of *Drosophila subobscura*: **(A)** richness (OTU number), **(B)** Shannon’s diversity, **(C)** phylogenetic diversity (Faith’s index), and **(D)** Pielou’s evenness. Flies of each sex were grouped into non-stressed (exposed to 21°C) and heat-stressed (exposed to 34°C) flies. Box plots show the median and interquartile range (IQR) and whiskers represent the 1.5*IQR. Symbols above box plots denote non-significant (ns) or significant differences between the non-stressed and heat-stressed flies obtained from linear models (**P < 0.01; ****P < 0.0001).

We analyzed the gut microbiota diversity of the flies exposed to a non-stressful temperature (21 C, non-stressed flies) and the flies exposed to a stressful thermal condition (34°C, heat-stressed flies). We found that OTU richness (Supplementary Table 2) was significantly lower in the heat-stressed flies than in the non-stressed flies and was not different between sexes, and we found a significant interaction between heat stress and sex (Table 2 and Figure 4A): thermal stress reduced the OTU number by 13.3% for female flies (Bonferroni *t* test: *P* = 0.002), whereas this reduction was 39.4% for male flies (Bonferroni *t* test: *P* = 6.2 × 10^–7^). On the other hand, Shannon diversity (Supplementary Table 2) showed non-significant effects associated with heat stress or sex, but a significant interaction between heat stress and sex was found (Table 2 and Figure 4B): non-stressed females harbor a lower diversity than the heat-stressed females (Bonferroni *t* test: *P* = 2.4 × 10^–10^), whereas non-stressed males showed higher diversity than the heat-stressed males (Bonferroni *t* test: *P* = 0.005). For the phylogenetic diversity (Supplementary Table 2), we found that heat-stressed flies showed a significantly lower phylogenetic diversity than the non-stressed flies, and no differences between sexes were detected, but we found a significant interaction between heat stress and sex (Table 2 and Figure 4C): non-stressed and heat-stressed females showed a similar phylogenetic diversity (Bonferroni *t* test: *P* = 0.18), whereas heat-stressed males harbor a lower phylogenetic diversity than the non-stressed females (Bonferroni *t* test: *P* = 5.5 × 10^–10^). Finally, Pielou’s evenness (Supplementary Table 2) showed a similar trend to Shannon diversity—a significant interaction between heat stress and sex (Table 2 and Figure 4D): non-stressed females harbor a lower diversity than the heat-stressed females (Bonferroni *t* test: *P* = 4.4 × 10^–10^), whereas non-stressed males showed higher diversity than the heat-stressed males (Bonferroni *t* test: *P* = 0.01).

**TABLE 2:** Results of the analysis of variance of the effects of heat stress (non-stressed and heat-stressed flies), sex (female and male flies), and its interaction on the bacterial diversity indices associated with the gut microbiota of *Drosophila subobscura*.

Diversity index	Heat stress	Sex	Interaction
OTU number (richness)	*F*_1,32_ = 124.81 *P* = 1.41 × 10^–12^	*F*_1,32_ = 3.11 *P* = 0.09	*F*_1,32_ = 33.43 *P* = 2.04 × 10^–6^
Shannon’s diversity (*H*′)	*F*_1,32_ = 0.41 *P* = 0.53	*F*_1,32_ = 3.43 *P* = 0.07	*F*_1,32_ = 63.19 *P* = 4.50 × 10^–9^
Phylogenetic diversity (Faith’s index)	*F*_1,32_ = 93.37 *P* = 5.21 × 10^–11^	*F*_1,32_ = 1.0 *P* = 0.33	*F*_1,32_ = 43.50 *P* = 1.96 × 10^–7^
Pielou’s evenness	*F*_1,32_ = 11 *P* = 0.75	*F*_1,32_ = 3.70 *P* = 0.06	*F*_1,32_ = 56.15 *P* = 1.57 × 10^–8^

**FIGURE 5:**
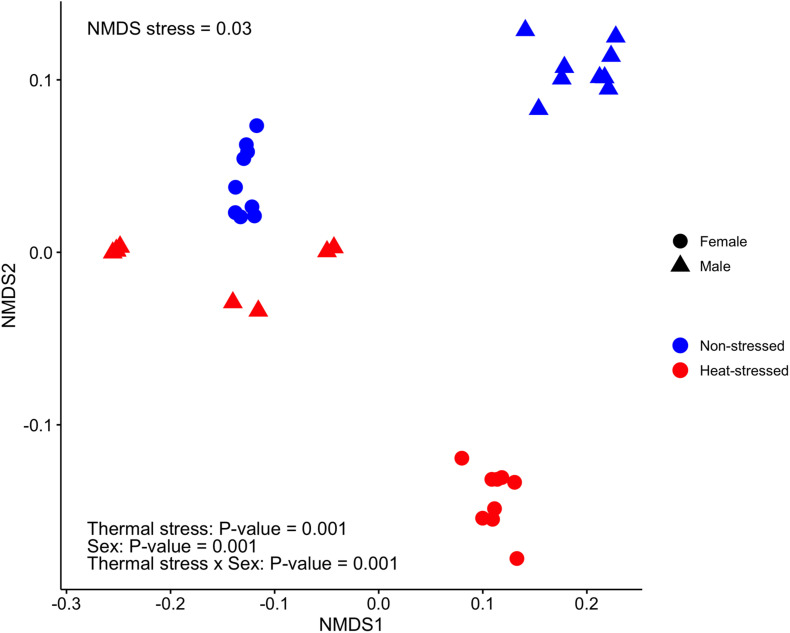
Bacterial community structure estimated for the gut microbiota of *Drosophila subobscura* using non-metric multidimensional scaling (NMDS) based on weighted UniFrac distances among samples. Flies of each sex were grouped into non-stressed (exposed to 21°C) and heat-stressed (exposed to 34°C) flies.

Finally, we found that the gut microbiota structure of *D. subobscura* was significantly affected by heat stress (*F*_1,32_ = 73.35, *P* = 0.001, *R*^2^ = 0.19) and sex (*F*_1,32_ = 33.48, *P* = 0.001, *R*^2^ = 0.09); we also found a significant interaction between heat stress and sex (*F*_1,32_ = 238.96, *P* = 0.001, *R*^2^ = 0.63). Heat stress had effects on the gut microbiota structure of female and male flies, with each group clustering separately (Figure 5).

## Discussion

Global warming impacts animals’ fitness, leading to an increased extinction risk in ectotherm species (Deutsch et al., 2008; Huey et al., 2012). The gut microbiota can contribute to host physiology leading to an increase of resistance to abiotic stressful conditions (Ferguson, 2018; Henry and Colinet, 2018). In the present work, we have studied the association between the gut microbiota and the thermal physiology of *D. subobscura*, representing the first characterization of the gut microbiota for this species. Our findings provide evidence that the gut microbiota influences heat tolerance and that heat stress modifies the gut microbiota at the taxonomical and diversity levels. These results demonstrate the sensitivity of the gut microbiota to transient heat stress, which can have negative impacts on host fitness.

### Gut Microbiota and Heat Tolerance

Several studies have evaluated the role of the gut microbiota on cold and heat tolerance in ectotherms, finding different results. For instance, Henry and Colinet (2018) found that the gut microbiota contributes to cold tolerance, but they found no differences in the heat tolerance between axenic and conventional flies of *D. melanogaster*. In the same line, Raza et al. (2020) found that the gut microbiota increases the tolerance to low temperatures in the dipteran *Bactrocera dorsalis*. On the other hand, a recent study found that the composition of the gut microbiota influences the heat tolerance of the western fence lizard (*Scleroporus occidentalis*), with a positive association between the abundance of the genus *Anaerotignum* (Firmicutes) and heat tolerance (Moeller et al., 2020). The different results about the role of the gut microbiota on heat tolerance could be due to the fact that heat tolerance depends on the methodology employed to measure it (Chown et al., 2009; Rezende et al., 2011; Castañeda et al., 2015), which can blur the physiological effects of different experimental treatments. To have a better approach to the thermal tolerance landscape (Rezende et al., 2014), we measured the heat tolerance at different temperatures from 35°C (mild thermal stress) to 38°C (intense thermal stress). Our results show that conventional flies tolerate better the high temperatures than do axenic flies, indicating that the gut microbiota positively influences the heat tolerance of *D. subobscura*. However, this positive effect of the gut microbiota on heat tolerance was only observed at the lowest assayed temperature (35°C): axenic flies tolerate this temperature for an average of 14.9 min, whereas conventional flies withstand it for 25.5 min. A plausible explanation for these findings is the impact of the gut microbiota on the host nutritional status (Ridley et al., 2012; Douglas, 2018a), which in turn determines the heat tolerance in ectotherms (Moghadam et al., 2018; Henry et al., 2019; Moeller et al., 2020; Semsar-Kazerouni et al., 2020). Similar to our findings, Semsar-Kazerouni et al. (2020) found that the effects of nutritional status on heat tolerance depended on the heat intensity, showing significant differences between fed and starved individuals exposed to mild heat stress. Then, if the gut microbiota impacts on the nutritional status, conventional flies can withstand longer heat stress than can axenic flies, but this difference can only be detected when there is enough time for the energy reserves to be used in costly resistance mechanisms associated with heat tolerance, such as heat shock proteins (Feder and Hofmann, 1999; Calabria et al., 2012; Hoekstra and Montooth, 2013). Heat shock proteins (HSPs) represent a key response to mitigating cellular damage during thermal stress (Sørensen et al., 2003; Calabria et al., 2012), and their expression can be induced as early as 15 min after exposure at 36°C in *D. melanogaster* (Hoekstra and Montooth, 2013). This evidence suggests that the gut microbiota could be enhancing the flies’ heat tolerance through higher HSP levels, which could also be supported because the gut microbiota can influence the expression of heat shock proteins in the gut epithelium of their hosts (Liu et al., 2014; Arnal and Lalle, 2016). Therefore, the next steps should involve studies on the interactions between the microbiota, nutritional status, and heat tolerance to understand the proximal mechanisms contributing to thermal tolerance in ectotherms.

### Gut Microbiota Composition

Recent studies have provided clear evidence of the impact of temperature on the gut microbiota of ectotherms [see Sepulveda and Moeller (2020) for a review]. In general, these studies have used thermal acclimation (i.e., >2 weeks) to evaluate changes in the gut microbiota composition, and it was found that, in warm temperatures, vertebrate ectotherms show a progressive decrease of bacteria belonging to Firmicutes (Bestion et al., 2017; Fontaine et al., 2018), whereas warm-temperature acclimation led to an increase of the relative abundance of Proteobacteria in invertebrate ectotherms (Berg et al., 2016; Moghadam et al., 2018; Horváthová et al., 2019). Here, we studied the effect of transient heat stress on the gut microbiota of *D. subobscura*, but we found a very different response of the bacterial composition when the flies were exposed to 34°C for 1 h. We found that the impact of heat stress led to an increase in abundance of 37.8% of the total OTUs, whereas 31.1% of OTUs decreased their abundances after heat stress. Interestingly, this short exposure to heat stress changed the gut microbiota composition differentially for each sex: heat stress induced a reduction in Firmicutes relative abundance and an increase in Actinobacteria abundance, whereas for males, we observed an increase of Firmicutes and a decline of Proteobacteria abundances. This sex-dependent response was also observed when we compared OTU abundances between the non-stressed and heat-stressed flies: 31 OTUs increased their abundances after heat stress in flies from both sexes, whereas only five OTUs showed higher abundances both in non-stressed female and male flies. Our findings suggest that temperature-induced changes in the gut microbiota of ectotherms can occur as fast as hours (present work), days (Sun et al., 2017), or weeks (Moghadam et al., 2018), which can explain the difference between our results and the expected increase of Proteobacteria in warm-acclimated ectotherms. Additionally, this difference can be explained by the fact that we analyzed the impact of temperature on the gut microbiota in both sexes, whereas other studies have assessed this impact using only males (Moghadam et al., 2018; Horváthová et al., 2019).

At the family level, we found that the gut microbiota of *D. subobscura* was dominated by acetic acid (Acetobacteraceae) and lactic acid (Lactobacillaceae and Leuconostoceae) bacteria, which is a common characteristic in *Drosophila* species (Douglas, 2018b). Regarding the effect of temperature on the bacterial family composition, *D. melanogaster* acclimated in warm conditions showed a higher abundance of *Acetobacter* bacteria (AAB) and a lower abundance of *Leuconostoc* bacteria (LAB) in comparison to cold-acclimated flies (Moghadam et al., 2018). Interestingly, this temperature-induced response of the gut microbiota composition under laboratory conditions matches wild populations, where low-latitude populations of *D. melanogaster* showed a higher AAB and a lower LAB abundance compared to high-latitude populations (Walters et al., 2020). Here, we found that AAB and LAB abundances changed with thermal stress, but these changes depended on the flies’ sex. In general, thermal stress reduced the Acetobacteraceae (AAB) and Lactobacillaceae (LAB) abundances, but the relative abundance of Leuconostoceae (LAB) increased in heat-stressed flies. The differences between *D. melanogaster* and *D. subobscura* can be explained as follows: 1) because they have traditionally been fed different diets, which is known to impact the gut microbiota composition (Jehrke et al., 2018; Obadia et al., 2018), or 2) just because they diverged around 40 million years ago (Gibbs and Matzkin, 2001), resulting in different evolutionary histories under different environmental contexts. Therefore, comparative studies are needed to understand the thermal plasticity of the gut microbiota in a wider range of *Drosophila* species.

### Gut Microbiota Diversity

Temperature also has important effects on the diversity and structure of the gut microbiota in ectotherms (Sepulveda and Moeller, 2020). In general, warm conditions lead to a decrease of OTU number (richness) and diversity of the gut microbiota in ectotherms (Bestion et al., 2017; Kokou et al., 2018; Li et al., 2018). Here, we found that heat stress induces a reduction in OTU number and that phylogenetic diversity decreased in heat-stressed flies, these effects being more important in males than in females. The similar response of richness and phylogenetic diversity is not surprising because, commonly, both diversity indices are highly correlated (Tôrres and Diniz-Filho, 2004). Conversely, Shannon diversity and evenness increased in heat-stressed females, but decreased in heat-stressed males, which is associated with the sex-specific changes in the abundances of some phyla in response to transient heat stress. Additionally, non-stressed females and heat-stressed males showed a more similar community structure compared to the other groups. These diversity and structure changes of the gut microbiota of *D. subobscura* in response to heat stress reflect a change in the dominant group: heat stress induced a reduction in Firmicutes abundance and an increase in Actinobacteria abundance, whereas for males, we observed an increase of Firmicutes and a decline of Proteobacteria abundances. Taxonomic-specific changes in the gut microbiota are common in ectotherms exposed to warm conditions, and it could be explained by the following: beyond the gut, bacteria have higher heat tolerance than eukaryotes (e.g., animal hosts), and they show high variability of their upper thermal limits (Storch et al., 2014). This suggests that some bacterial species can tolerate better direct and/or indirect effects of heat stress, including the production of reactive oxygen species by hosts as a response to heat stress (Lian et al., 2020). However, our study had some limitations in explaining the proximal causes of the changes in bacterial abundances, and future steps should be focused on exploring the resistance mechanisms in members of the gut microbiota.

## Conclusion

Temperature induces changes in the gut microbiota of ectotherms, regardless of how long organisms have been exposed to warm conditions. Here, we demonstrated that these changes are different for both sexes, and future studies should assess the sexual dimorphism in gut microbiota responses to abiotic and biotic factors. These changes in the gut microbiota have consequences on the physiological mechanisms such as thermal resistance, which can impact host fitness, population risk extinction, and the vulnerability of ectotherms to current and future climatic conditions. Research about the role of the gut microbiota on the adaptive response to climate change is a new venue, and future research needs to balance mechanistic approaches in order to understand host–microbiota interactions and holistic approaches in order to know the role of the gut microbiota in the ecology and evolution of ectotherms.

## Data Availability Statement

The datasets analyzed in this study can be found in https://figshare.com/s/07258a71c3074fa59b1e. Amplicon sequences analyzed in this study were deposited in MG-RAST (https://www.mg-rast.org/linkin.cgi?project=mgp98467).

## Ethics Statement

Ethical review and approval was not required for the animal study because the manuscript presents results of research on invertebrate animals (Drosophila).

## Author Contributions

AJ performed the experiments, analyzed the dataset, and approved the final version of the manuscript. LC conceived the original idea, designed the experiments, conducted bioinformatic and statistical analyses, provided funds for all experiments, and wrote the manuscript. Both authors contributed to the article and approved the submitted version.

## Conflict of Interest

The authors declare that the research was conducted in the absence of any commercial or financial relationships that could be construed as a potential conflict of interest.
