# Supplementary material for: Gut Microbiota of Drosophila subobscura Contributes to Its Heat Tolerance and Is Sensitive to Transient Thermal Stress
Source: Front Microbiol. 2021 May 6;12:654108. doi: 10.3389/fmicb.2021.654108 (PMC8137359; doi:10.3389/fmicb.2021.654108)
Supplement: Supplementary file 1 [file Data_Sheet_1.docx]

Supplementary Material

Gut microbiota of *Drosophila subobscura* contributes to its heat tolerance and is sensitive to transient thermal stress

**Angélica Jaramillo, Luis E. Castañeda**

**Supplementary Figure 1**. Rarefaction plots indicating OTU number and sequencing depth after rarefaction at 12,000 sequences. Samples were taken from non-stressed females, non-stressed males, heat-stressed females, and heat-stressed males flies of *D. subobscura*.

**Supplementary Figure 2.** Knockdown curves of axenic and conventional flies of *D. subobscura* assayed at 35 ºC (A), 36 ºC (B), 37 ºC (C), and 38 ºC (D). Curves were estimated using a Kaplan-Meier model, and shaded areas indicate 95% confidence interval of each survival curve. Curves were compared using G-rho family test (log-rank test) and resulting P-values are shown.

**
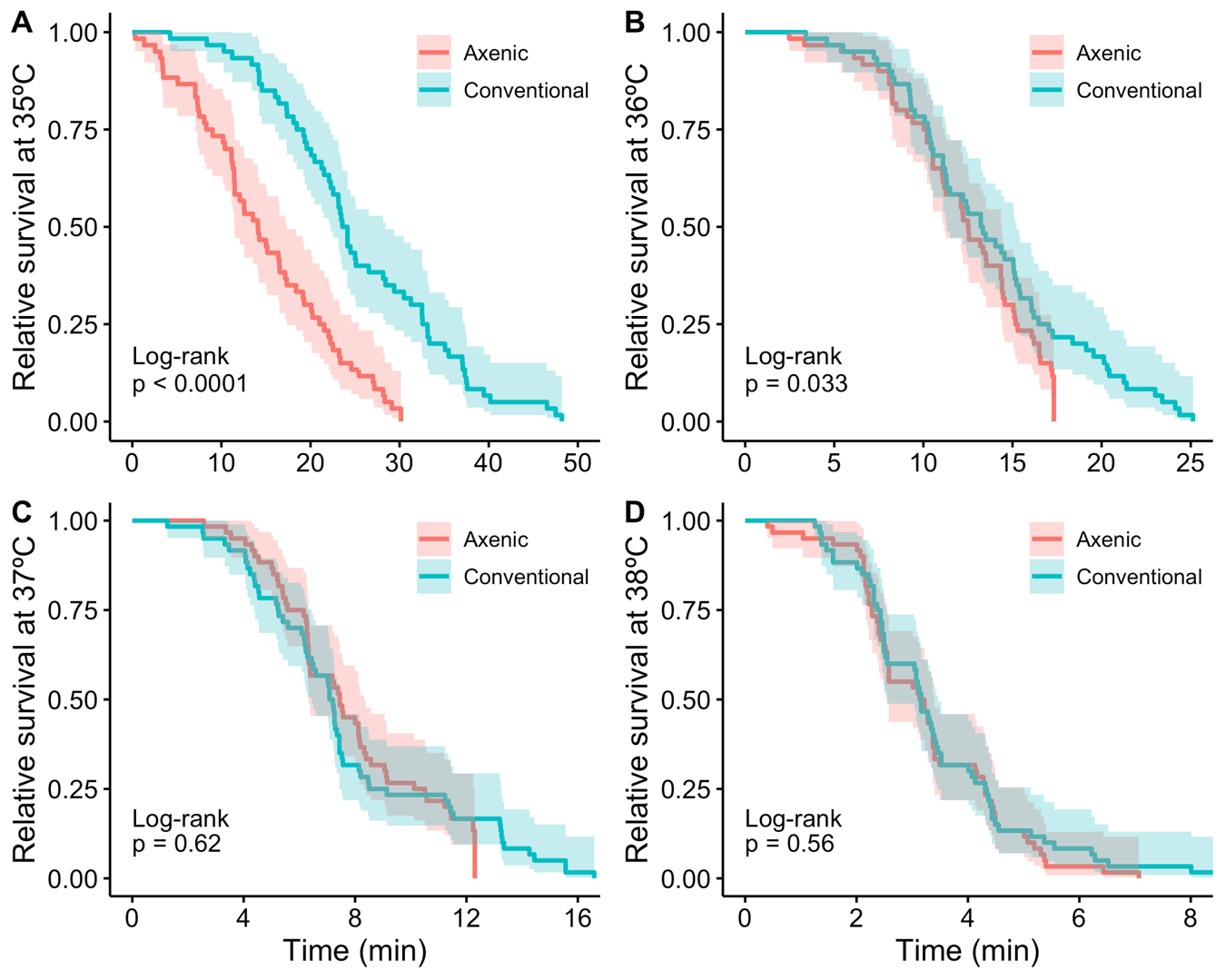
**

**Supplementary Figure 3**. Relative abundances of the phyla (A) Actinobacteria, (B) Bacteroidetes, (C) Proteobacteria, and (D) Firmicutes found in the gut of *D. subobscura*. Flies of each sex were grouped into non-stressed flies (exposed to 21ºC) and heat-stressed flies (exposed to 34ºC). Box plots show median and interquartile range (IQR) and whiskers represent the 1.5*IQR. Symbols above boxplots denote non-significant (ns) or significant differences between non-stressed and heat-stressed flies obtained from generalized-linear models (*: *P* < 0.05; **: *P* < 0.01; ***: *P* < 0.001; ****: *P* < 0.0001).


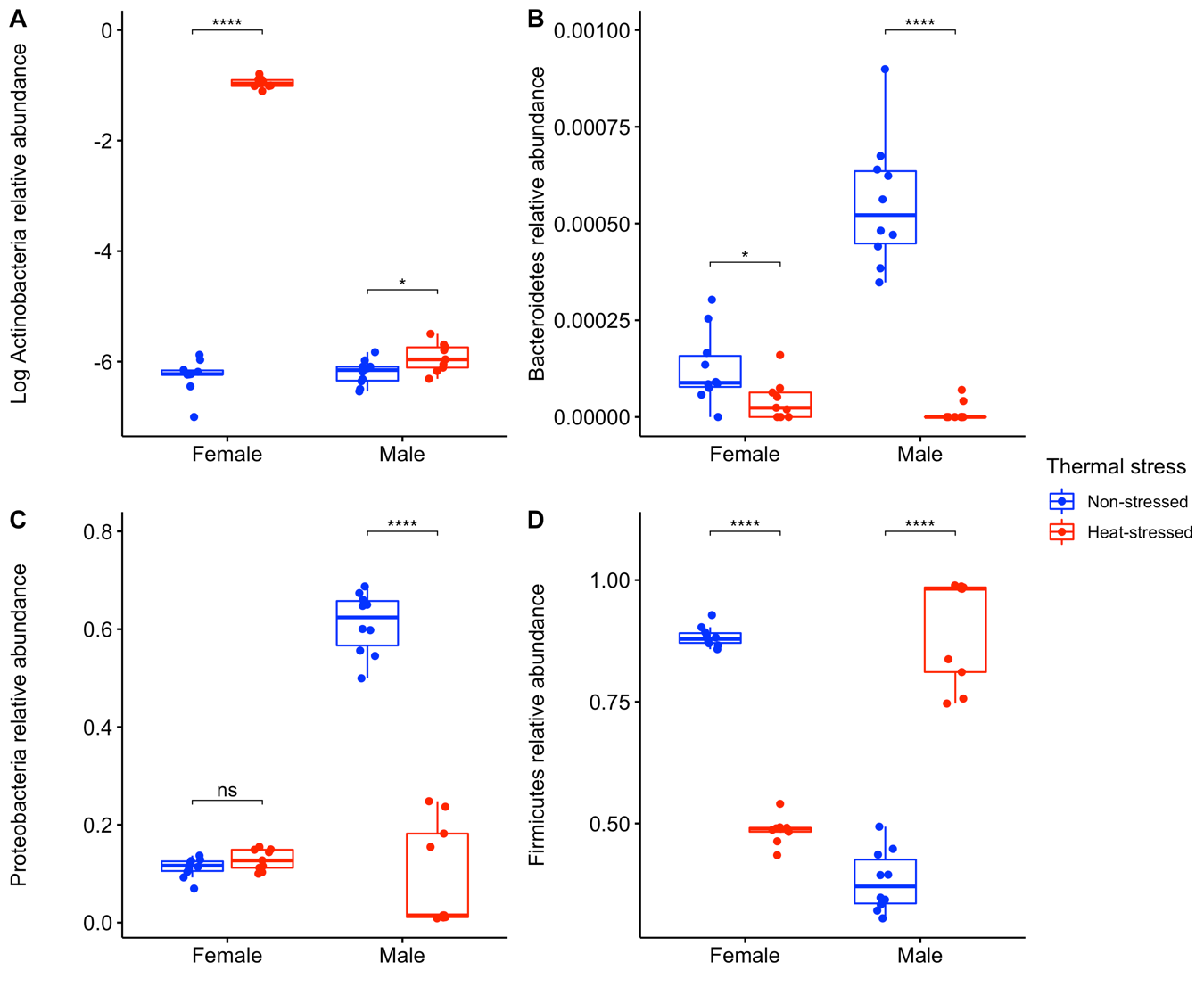


**Supplementary Figure 3**. Relative abundances of the bacterial families (A) Acetobacteraceae, (B) Lactobacillaceae, and (C) Leuconostoceae found in the gut of *D. subobscura*. Flies of each sex were grouped into non-stressed flies (exposed to 21ºC) and heat-stressed flies (exposed to 34ºC). Box plots show median and interquartile range (IQR) and whiskers represent the 1.5*IQR. Symbols above boxplots denote non-significant (ns) or significant differences between non-stressed and heat-stressed flies obtained from generalized-linear models (*: *P* < 0.05; **: *P* < 0.01; ***: *P* < 0.001; ****: *P* < 0.0001).


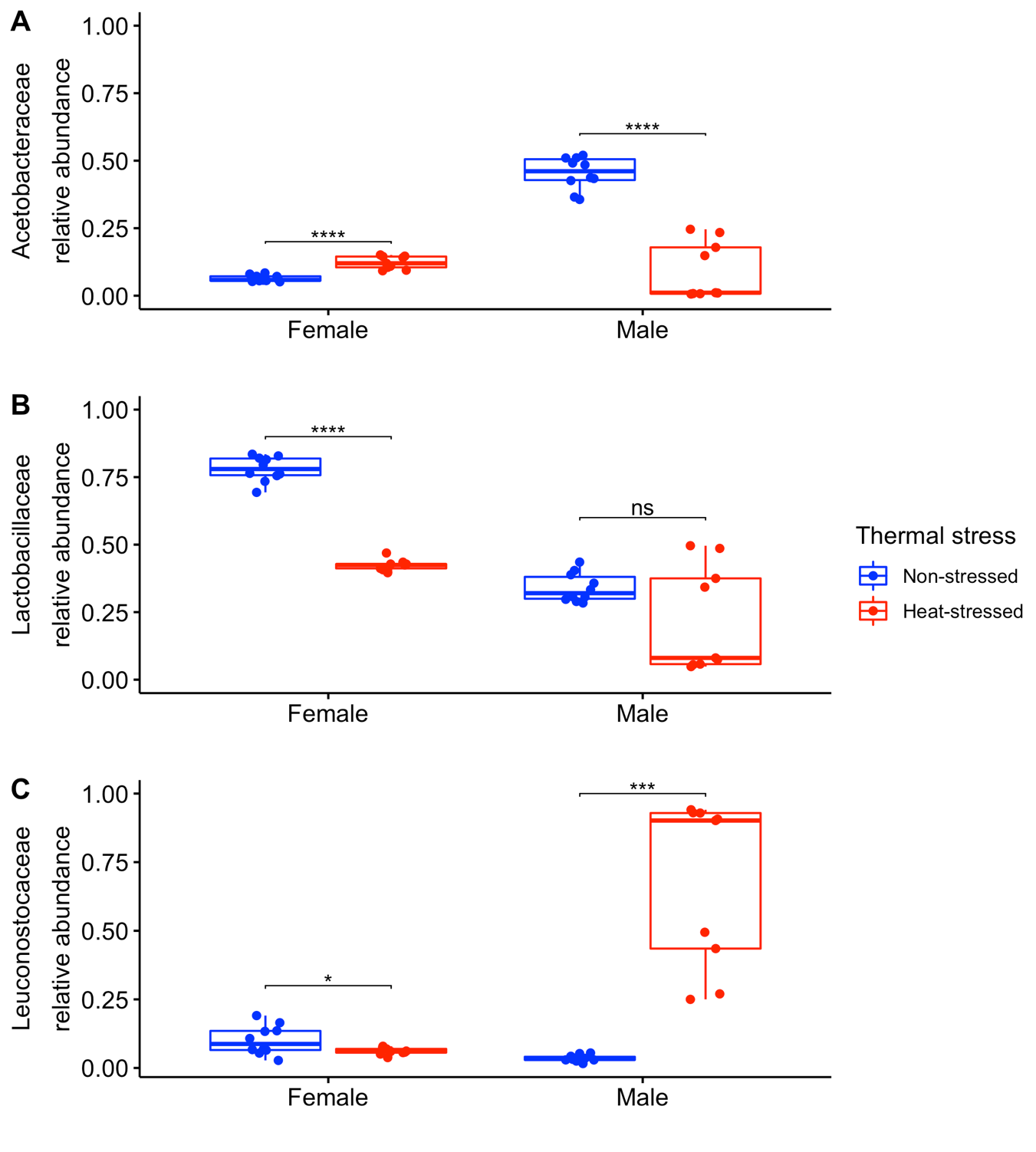


**Supplementary Table 1**. Modified 341F and 805F primers for bacterial 16S rRNA gene used in this study. Each primer contains: a linker sequence to bind amplicons to the Nextera XT DNA indexes (black letters); a 12 bp barcode sequence to multiplex samples (red letters); a 0 to 5 bp "heterogeneity spacer" (blue letters), and 16S rRNA gene universal primers (green letters).

| **Primer** | **Illumina linker sequence – 12 bp barcode sequence – Heterogeneity spacer – 16S rRNA primer** |
| --- | --- |
| 16S_For_A | TCGTCGGCAGCGTCAGATGTGTATAAGAGACAG – CCTAAACTACGG – CCTACGGGNGGCWGCAG |
| 16S_For_B | TCGTCGGCAGCGTCAGATGTGTATAAGAGACAG – TGCAGATCCAAC –T – CCTACGGGNGGCWGCAG |
| 16S_For_C | TCGTCGGCAGCGTCAGATGTGTATAAGAGACAG – CCATCACATAGG – GT – CCTACGGGNGGCWGCAG |
| 16S_For_D | TCGTCGGCAGCGTCAGATGTGTATAAGAGACAG – GTGGTATGGGAG – CGA – CCTACGGGNGGCWGCAG |
| 16S_For_E | TCGTCGGCAGCGTCAGATGTGTATAAGAGACAG – ACTTTAAGGGTG – ATGA – CCTACGGGNGGCWGCAG |
| 16S_For_F | TCGTCGGCAGCGTCAGATGTGTATAAGAGACAG – GAGCAACATCCT – TGCGA – CCTACGGGNGGCWGCAG |
| 16S_Rev_A | GTCTCGTGGGCTCGGAGATGTGTATAAGAGACAG – CCTAAACTACGG – GACTACHVGGGTATCTAATCC |
| 16S_Rev_B | GTCTCGTGGGCTCGGAGATGTGTATAAGAGACAG – TGCAGATCCAAC – A – GACTACHVGGGTATCTAATCC |
| 16S_Rev_C | GTCTCGTGGGCTCGGAGATGTGTATAAGAGACAG – CCATCACATAGG – TC – GACTACHVGGGTATCTAATCC |
| 16S_Rev_D | GTCTCGTGGGCTCGGAGATGTGTATAAGAGACAG – GTGGTATGGGAG – CTA – GACTACHVGGGTATCTAATCC |
| 16S_Rev_E | GTCTCGTGGGCTCGGAGATGTGTATAAGAGACAG – ACTTTAAGGGTG – GATA – GACTACHVGGGTATCTAATCC |
| 16S_Rev_F | GTCTCGTGGGCTCGGAGATGTGTATAAGAGACAG – GAGCAACATCCT – ACTAC – GACTACHVGGGTATCTAATCC |

**Supplementary Table 2.** Information for each sample about the thermal stress and sex of Drosophila subobscura included in this study. Number of sequences, removal after rarefaction at 12,000 sequences, and richness (OTU number), Shannon diversity, and phylogenetic diversity estimates for each sample are provided. NA means non-available estimate because sample was removed after rarefaction (see Material and Methods section).

| **Sample ID** | **Sex** | **Thermal stress** | **Sequences** | **Sample removed after rarefaction** | **Richness**  **(OTU number)** | **Shannon**  **diversity** | **Phylogenetic**  **diversity** |
| --- | --- | --- | --- | --- | --- | --- | --- |
| 1FDRD | Female | Heat-stressed | 746 | Yes | NA | NA | NA |
| 2FDRD | Male | Heat-stressed | 860 | Yes | NA | NA | NA |
| 2FBRB | Male | Non-stressed | 5748 | Yes | NA | NA | NA |
| 1FBRB | Female | Non-stressed | 6596 | Yes | NA | NA | NA |
| 2FCRC | Female | Heat-stressed | 12496 | No | 90 | 2.0783283 | 5.14382 |
| 1FCRC | Male | Heat-stressed | 13042 | No | 62 | 0.7040019 | 4.18603 |
| 1FARA | Male | Non-stressed | 15864 | No | 105 | 1.9065749 | 6.05509 |
| 2FARA | Female | Non-stressed | 17341 | No | 83 | 1.203535 | 4.47469 |
| 2FBRD | Male | Heat-stressed | 24885 | No | 72 | 1.5965272 | 4.23032 |
| 2FCRD | Male | Heat-stressed | 28514 | No | 74 | 1.6631817 | 4.33094 |
| 2FCRB | Male | Non-stressed | 33992 | No | 99 | 2.0364269 | 5.64249 |
| 2FCRA | Female | Non-stressed | 35376 | No | 88 | 1.26127 | 4.64091 |
| 2FDRC | Female | Heat-stressed | 38526 | No | 77 | 1.9712162 | 4.80308 |
| 2FBRC | Female | Heat-stressed | 39676 | No | 87 | 2.045393 | 4.62237 |
| 2FARC | Female | Heat-stressed | 40144 | No | 78 | 2.0240818 | 4.80176 |
| 1FCRA | Male | Non-stressed | 41559 | No | 103 | 1.8614536 | 5.54767 |
| 2FERC | Female | Heat-stressed | 41693 | No | 81 | 2.0365678 | 4.91643 |
| 2FDRB | Male | Non-stressed | 42681 | No | 99 | 1.8427923 | 5.61006 |
| 2FBRA | Female | Non-stressed | 44190 | No | 88 | 1.2241704 | 4.83322 |
| 2FARD | Male | Heat-stressed | 45337 | No | 75 | 1.7221588 | 4.37624 |
| 2FERB | Male | Non-stressed | 45609 | No | 99 | 1.8698738 | 5.42088 |
| 1FDRC | Male | Heat-stressed | 45911 | No | 56 | 0.6206609 | 3.63735 |
| 2FERA | Female | Non-stressed | 46086 | No | 99 | 1.3249281 | 5.09436 |
| 2FDRA | Female | Non-stressed | 46127 | No | 98 | 1.2309471 | 5.04824 |
| 1FERD | Female | Heat-stressed | 47339 | No | 82 | 2.0531454 | 4.62203 |
| 1FERB | Female | Non-stressed | 48321 | No | 86 | 1.4479118 | 4.73222 |
| 1FBRC | Male | Heat-stressed | 48350 | No | 53 | 0.5368444 | 3.75234 |
| 1FARC | Male | Heat-stressed | 49183 | No | 52 | 0.5356601 | 4.04646 |
| 1FERA | Male | Non-stressed | 49422 | No | 101 | 1.9705597 | 5.80055 |
| 1FCRD | Female | Heat-stressed | 49884 | No | 79 | 2.044334 | 4.64284 |
| 1FERC | Male | Heat-stressed | 50290 | No | 54 | 0.4892269 | 3.95857 |
| 1FBRD | Female | Heat-stressed | 50902 | No | 73 | 1.9746317 | 4.1562 |
| 1FDRB | Female | Non-stressed | 51134 | No | 98 | 1.366587 | 5.00307 |
| 2FARB | Male | Non-stressed | 51351 | No | 107 | 1.9728177 | 5.8345 |
| 1FCRB | Female | Non-stressed | 53051 | No | 99 | 1.3970142 | 5.49794 |
| 1FDRA | Male | Non-stressed | 53163 | No | 100 | 1.8445902 | 5.66431 |
| 1FARD | Female | Heat-stressed | 53901 | No | 69 | 2.0003884 | 3.73369 |
| 1FBRA | Male | Non-stressed | 54842 | No | 95 | 1.8485612 | 5.60939 |
| 2FERD | Male | Heat-stressed | 55058 | No | 64 | 1.6865227 | 3.8092 |
| 1FARB | Female | Non-stressed | 59210 | No | 87 | 1.3002729 | 4.92085 |
